# Double drugging of prolyl-tRNA synthetase provides a new paradigm for anti-infective drug development

**DOI:** 10.1371/journal.ppat.1010363

**Published:** 2022-03-25

**Authors:** Yogavel Manickam, Nipun Malhotra, Siddhartha Mishra, Palak Babbar, Abhishek Dusane, Benoît Laleu, Valeria Bellini, Mohamed-Ali Hakimi, Alexandre Bougdour, Amit Sharma

**Affiliations:** 1 Molecular Medicine–Structural Parasitology Group, International Centre for Genetic Engineering and Biotechnology (ICGEB), New Delhi, India; 2 ICMR-National Institute of Malaria Research (NIMR), New Delhi, India; 3 Academy of Scientific and Innovative Research (AcSIR), Ghaziabad, India; 4 Medicines for Malaria Venture (MMV), International Center Cointrin (ICC), Geneva, Switzerland; 5 Institute for Advanced Biosciences (IAB), Team Host-Pathogen Interactions and Immunity to Infection, INSERM U1209, CNRS UMR5309, Université Grenoble Alpes, Grenoble, France; Heidelberg University, GERMANY

## Abstract

Toxoplasmosis is caused by *Toxoplasma gondii* and in immunocompromised patients it may lead to seizures, encephalitis or death. The conserved enzyme prolyl-tRNA synthetase (PRS) is a validated druggable target in *Toxoplasma gondii* but the traditional ‘single target–single drug’ approach has its caveats. Here, we describe two potent inhibitors namely halofuginone (HFG) and a novel ATP mimetic (L95) that bind to *Toxoplasma gondii* PRS simultaneously at different neighbouring sites to cover all three of the enzyme substrate subsites. HFG and L95 act as one triple-site inhibitor in tandem and form an unusual ternary complex wherein HFG occupies the 3’-end of tRNA and the L-proline (L-pro) binding sites while L95 occupies the ATP pocket. These inhibitors exhibit nanomolar IC_50_ and EC_50_ values independently, and when given together reveal an additive mode of action in parasite inhibition assays. This work validates a novel approach and lays a structural framework for further drug development based on simultaneous targeting of multiple pockets to inhibit druggable proteins.

## Author summary

Among infectious diseases, parasitic diseases are a major cause of death and morbidity. *Toxoplasmosis* is caused by an infection of the apicomplexan parasite *Toxoplasma gondii*. In immunocompromised patients *Toxoplasmosis* may lead to seizures, encephalitis or death. Novel therapeutics for human parasites are constantly needed. In recent years, the aminoacyl-tRNA synthetase (aaRS) enzyme family has been validated as a drug target for several parasitic infections. The *Toxoplasma gondii* prolyl-tRNA synthetase inhibitor halofuginone (HFG) has been validated earlier but here we show that an ATP-mimic called L95 is a potent inhibitor and can bind to the target enzyme in the presence of HFG. Thus, the two inhibitors described in this study simultaneously occupy all three natural substrate (ATP, L-amino acid and 3’-end of tRNA) binding pockets and thereby inhibit the enzyme leading to parasite death. This unprecedented double drugging of a pathogen enzyme may delay resistance mutation generation and this approach opens the path to multi-drugging of validated parasite proteins.

## Introduction

Management of infectious diseases continues to be a huge priority for the global medical community as they comprise nearly half of the major global health concerns [1]. Amongst them, apicomplexan parasites like *Plasmodia* and *Toxoplasma* that cause malaria and toxoplasmosis are causes of morbidity and mortality worldwide [2,3]. *Toxoplasma gondii* (*Tg*) is an obligate intracellular parasitic protozoan which causes toxoplasmosis. It is one of the most common parasites in developed countries with a serological infection rate of up to 30–50% [4]. In humans, active toxoplasmosis can be treated with a combination of drugs such as pyrimethamine and sulfadiazine along with folinic acid [5]. Considering the likely development of drug resistance against available therapeutics, there is a continued need for identifying novel targeting strategies against this parasitic infection.

Aminoacyl-tRNA synthetases (aaRSs) are highly conserved and essential enzymes responsible for feeding charged tRNA molecules into the protein synthesis machinery [6,7]. The active site of aaRSs has three closely located sub-sites for natural substrates L-amino acid (aa), ATP and 3’-end tRNA [6]. Inhibiting these enzymes stalls protein translation. By virtue of this, several aaRSs have been recognized as druggable targets for infectious and human diseases [8–11]. In recent years, inhibitors targeting their active and allosteric sites have been reported [12–15]. The active site inhibitors were further grouped based on their binding modes such as single (any one of the substrate sub-sites inhibited) [16–19], dual (overlapping of two substrate subsites [20–22] or substrate subsite with an auxiliary pocket [23,24]), triple or multi-site (covering all substrate subsites) [25], while the non-aminoacylation site inhibitors were grouped into editing, allosteric and non-translational function-site inhibitors [26–29]. Prolyl-tRNA synthetases (PRS) charge tRNA molecules with L-pro for protein translation. Over the last decade, parasite- and host-encoded PRSs have been extensively studied using febrifugine (FF) and its derivatives including halofuginone (HFG) that block both the L-pro and the 3’-end of the tRNA binding pockets [20–21,30–32]. Hewitt et al. in 2017 showed that glyburide and the ligand TCMDC124506 can target the PRS enzyme allosterically [27]. Recent advancements towards ATP mimetics targeting the ATP pocket of PRSs have opened a new window towards drug development [33–36]. A partner group recently published a series of inhibitors that targets the ATP pockets of both malaria and host PRSs [37].

The simultaneous targeting of distinct catalytic sites of a single target using a cooperative ligand binding approach has also been explored recently. Although structurally incomplete and poorly understood, these discoveries highlight the importance of multivalent targeting strategies in modulating the efficacy of druggable hits [38–42].

**Fig 1:**
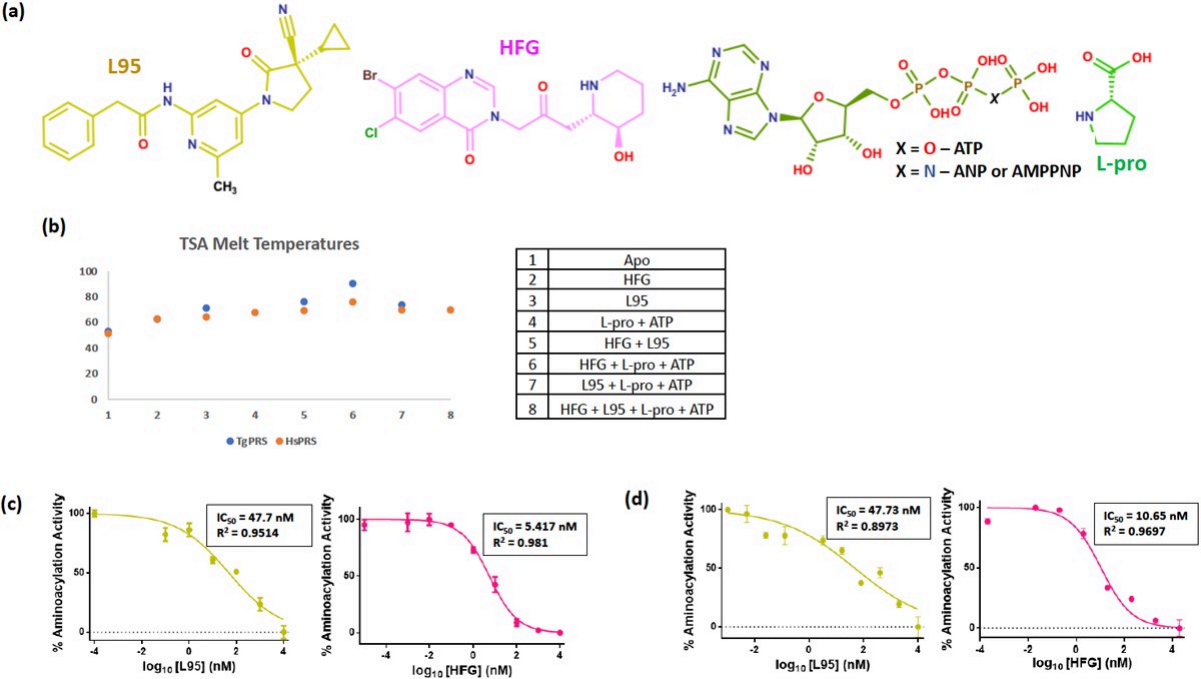
Biochemical binding and inhibition profiles of HFG and L95. (a) Chemical structures of L95, halofuginone (HFG), L-proline and ATP/ANP (Non hydrolysable ATP analog). (b) TSA melt temperatures of *Tg*PRS and *Hs*PRS (at 1 μM) in the presence of inhibitors alone or along with substrate. (c) *Tg*PRS aminoacylation activity inhibition assays in the presence of HFG and L95. For these the concentration for compounds ranged from 50 to 0.005 μM. (d) *Hs*PRS aminoacylation activity inhibition assays in the presence of HFG and L95. For these the concentration for compounds ranged from 50 to 0.005 μM.

In this study, we examined a pyrrolidine-based ATP mimetic–PubChem ID SCHEMBL22505739 (hereafter labelled as L95, Figs 1A and S1) and validated it as an ATP mimetic inhibitor against *Toxoplasma gondii* PRS (*Tg*PRS) enzyme. Moreover, we hypothesized that L95 in concert with HFG can bind to and inhibit *Tg*PRS as the two ligands reside in close proximity but different substrate pockets. We validated our hypothesis via biochemical, structural and cellular evidence. Here, we present high-resolution crystal structures of two ternary complexes; a single liganded *Tg*PRS-L-pro-L95 and concurrently a bound double liganded *Tg*PRS-HFG-L95. Our work serves as proof for simultaneous targeting of PRS enzyme with two ligands and this study lays a framework for new drug development that focuses on a novel approach to counter drug resistance in major infectious diseases like malaria and toxoplasmosis.

## Results

### Two inhibitors HFG and L95 binding simultaneously to the target *Tg*PRS enzyme

The binding potencies of the inhibitors (L95 and HFG) in the presence or absence of substrates (L-pro and ATP) were evaluated against recombinant *Tg*PRS enzyme using a fluorescence-based thermal shift assay (TSA). The ligand (inhibitors/substrates) binding usually helps to stabilize the enzyme due to enzyme-ligand interactions, resulting in an increase of the melting temperature (T_m_) of the target protein during the thermal denaturation process [43]. The ΔT_m_ was calculated based on the difference between the T_*m*_ values of *Tg*PRS with and without ligands. The T_m_ of *Tg*PRS-apo is 52.3°C. In the presence of L95 alone, the T_m_ shifts to a higher temperature by ΔT_m_ of 17.8°C, whereas in the additional presence of L-pro, the thermal stability of *Tg*PRS enzyme improves significantly resulting in ΔT_m_ value of 20.7°C suggesting that the inhibitor L95 is binding to *Tg*PRS in an L-pro-dependent manner (Figs 1B and S2). The higher ΔT_m_ of L95 in the presence of L-pro implies that induced conformational changes may stabilize the ternary complex of *Tg*PRS-L-pro-L95 and suggests a cooperative binding mode of L95 with L-pro. Similarly, the measured ΔT_m_ for HFG alone and HFG with ATP were 8.1°C and 37.6°C respectively, which is similar to previously reported values with the ATP facilitating HFG integration within the active site of the enzyme [20,21,44,45].

Next, we validated that the inhibitor L95 occupies the ATP binding pocket of *Tg*PRS more effectively in the presence of L-pro (Fig 1B, compare L95 with L95+L-pro). Further, we hypothesized the two drugs HFG and L95 occupying the three pockets together: L-pro, 3’-end of tRNA and ATP within the active site. For this, both HFG and L95 were added to *Tg*PRS simultaneously and the ternary complex of *Tg*PRS-HFG-L95 showed a ΔT_m_ of 23.6°C. (Fig 1B). The similar ΔT_m_ values of *Tg*PRS-HFG-L95 and *Tg*PRS-L-pro-L95 suggest that the association of *Tg*PRS with L95 in the presence of L-pro is quite similar to its binding with L95 in presence of HFG. However, the slightly higher ΔT_m_ value for *Tg*PRS-HFG-ATP indicates that ATP (natural substrate) provides more stability than L95.

### PRS enzyme inhibition by L95

The enzymatic activity of recombinant proteins *Tg*PRS and *Hs*PRS were evaluated in the presence of either HFG or L95 by performing aminoacylation assays. The first part of the aminoacylation reaction where the aminoacyl-adenylate complex is formed was studied in the presence of these inhibitors. Here, the aminoacyl-adenylate complex is formed with the release of pyrophosphate (PPi) which can be indirectly measured by a malachite green assay that uses the pyrophosphatase-induced conversion of PPi into inorganic phosphate (Pi). For the enzyme inhibition assay, concentration ranges of 50 to 0.005 μM were used for HFG and L95. The measured half-maximal inhibitory concentration (IC_50_) values computed for *Tg*PRS in the presence of individual inhibitors HFG and L95 were 5.41 nM and 47.7 nM respectively (Fig 1C). The measured IC_50_ value for HFG against *Tg*PRS is very similar to our previous reports [20]. For *Hs*PRS, the IC_50_ values computed for HFG and L95 were 10.65 nM and 47.7 nM respectively. The high potency of these inhibitors is evident from their nanomolar IC_50_ values.

### Structural features of single (L95) and concurrently double liganded (HFG-L95) *Tg*PRS enzyme

**Fig 2:**
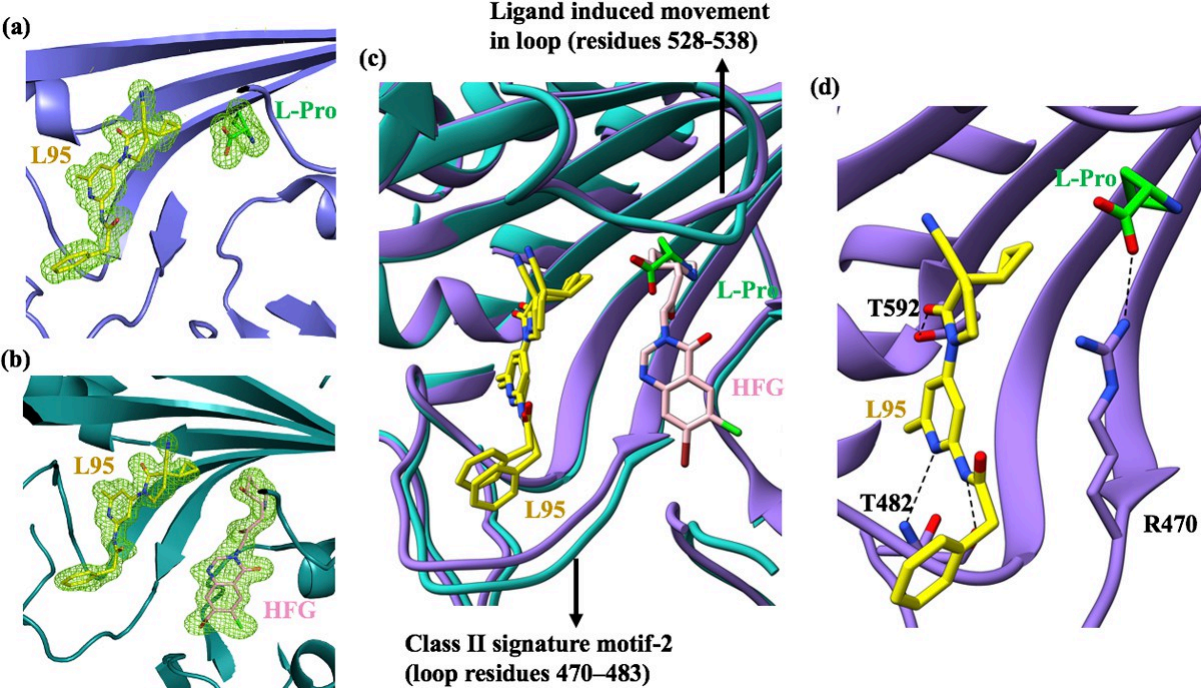
Crystal Structure summary of HFG and L95 bound to *Tg*PRS. (a and b) Difference Fourier map (Fo-Fc) of the bound ligands in the aminoacylation site of *Tg*PRS. The Fo-Fc maps are contoured at 3 σ and shown as green mesh representations. Ligands L-pro (green) L95 (yellow), HFG (pink) are shown as sticks. (c) Superposition of *Tg*PRS-L-pro-L95 and *Tg*PRS-HFG-L95 complexes showing the ligand induced movement of loops. (d) The interactions between *Tg*PRS and bound L-pro-L95. The salt bridge interaction with L-pro and interacting residues with L95 are shown as sticks. The hydrogen bonds are shown as black dashed lines.

**Fig 3:**
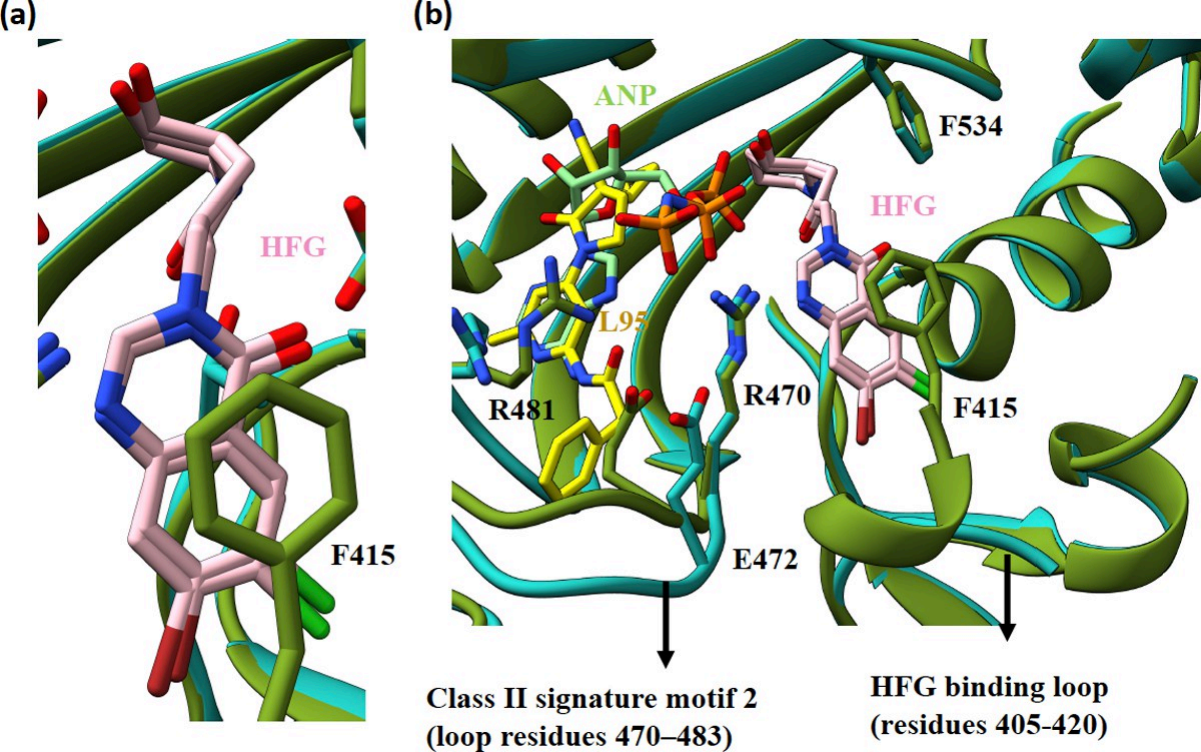
Structural comparison of HFG-ANP and HFG-L95 bound *Tg*PRSs. (a and b) Superposition of *Tg*PRS-HFG-L95 (PDB id 7EVU) (cyan) and *Tg*PRS-HFG-ANP (PDB id 5XIQ) (olive) complexes showing the bound pose of ligand HFG and ligand induced movement of loops.

To understand the binding mechanism of the single and double liganded *Tg*PRSs, we determined two co-crystal structures of *Tg*PRS in complex with L95 and L-pro (*Tg*PRS-L-pro-L95) (PDB id 7EVV) and another in complex with HFG and L95 (*Tg*PRS-HFG-L95) (PDB id 7EVU) at a resolution of 1.7 Å (Figs 2 and 3 and Table 1). Difference Fourier electron density maps confirmed the presence of L95 in the ATP pocket (Fig 2A). There is one monomer per asymmetric unit in the C2 space group of the *Tg*PRS-L-pro-L95 ternary complex, while the biologically active dimer has crystallised in the P1 space group for the concurrently double liganded *Tg*PRS enzyme (Figs 2B and S3). Superimposition of two chains in the *Tg*PRS-HFG-L95 complex shows a high degree of similarity as evidenced by the overall mean RMSD values of 0.28 Å for 480 C^〈^-atoms. Therefore, for all structural descriptions, here on we have only considered chain A.

**Table 1:** Summary of data collection and refinement statistics. Values in parentheses are for the highest resolution shell.

	*Tg*PRS-L-pro-L95	*Tg*PRS-HFG-L95
PDB code	7EVV	7EVU
**Data collection**		
Beamline	PROXIMA-1 (PX1)	I03, DLS
Wavelength (Å)	0.979	0.976
Detector type	EIGER X 16M	Eiger2 XE 16M
Crystal-to-detector distance (mm)	270.6	
Oscillation (°)	0.1	0.1
Exposure (s)	0.5	0.02
No. of images	3600	3600
Software used for data processing	*XDS*	*XIA2/DIALS*
Space group	C2	P1
Cell dimensions		
a, b, c (Å)	111.35, 75.20, 74.74;	70.75, 70.83, 76.04;
α, β, γ (°)	90.0, 110.7, 90.0	92.16, 101.44, 115.61
Resolution (Å)	41.85–1.70 (1.71–1.70)	61.97–1.70 (1.73–1.70)
Rmeas (%)	8.9 (60.2)	11.3 (252.2)
I/σI	13.3 (2.9)	6.0 (1.3)
Completeness (%)	99.1 (95.7)	96.9 (89.3)
Redundancy	2.2 (2.3)	3.5 (3.7)
CC1/2 (%)	99.2 (86.8)	98.9 (31.4)
No. of unique reflections	62494 (9672)	138636 (6454)
**Refinement**		
Resolution (Å)	31.52–1.7 (1.73–1.7)	59.62–1.7 (1.71–1.7)
No. of reflections /test set	62474/3122	138595 / 6805
Rwork/ Rfree (%)	15.3/18.1	19.8/23.0
No. of protein residues	490	484 + 481
No. atoms		
Protein /Water	4040/287	7895/556
Ligand (non-H atoms) L95+ L-pro/HFG+L95	36 (28 + 8)	104 (48 + 56)
Ions /Solvent molecules^#^	1/16	2/42
Average B-factors (Å^2^)		
Protein /Water	31.6/37.9	35/45.4
Ligand (non-H atoms) L95- L-pro/HFG + L95	21.7 (23.5/19.8)	27 (27.1/28.8)
Cl ion / MPD/MES	77.4/42.6	33.5
R.m.s deviations		
Bond lengths (Å)	0.018	0.016
Bond angles (°)	1.508	1.400
Ramachandran plot		
Favoured/Allowed (%)	97.9 / 2.1	98 / 2
Clash score	3.1	3
Disordered Regions	408–415, 787–788	408–416, 784–790

^#^ Ions: Cl/Br and Solvent molecules: MPD, MES, EDO, IMD

Superposition of the ATP pocket of both L95 bound structures showed that L95 adopts a similar but not identical conformation with minute deviations in the phenylacetamide moiety (Fig 2C). The class II signature motifs 2 (residues 460–494) and 3 (residues 585–603) of *Tg*PRS form multiple interactions with L95. The inhibitor L95 is stabilized mainly by three direct and three water-mediated hydrogen bonds. The three direct hydrogen bonds are: (1) 6-methylpyridine N1 atom with main-chain N atom of Thr482 (2) phenylacetamide N-atom with main-chain O of Thr482 and (3) oxo-pyrrolidine keto O atom with side-chain OG1 atom of Thr592 (Figs 2D and S4). The hydrogen-bonding interactions between the hinge region of L95 and the protein main chain is very similar to that observed in Tyk2 inhibition [46]. The 6-methylpyridine ring is sandwiched by Phe485 via π-π stacking interaction (~3.9 Å) and Arg594 via cation-π interaction (S5 Fig). Superposition of the ligand complexes HFG-L95 (PDB id 7EVU) and HFG-ANP (non-hydrolysable ATP analog) (PDB id 5XIQ) showed that the inhibitor HFG adopts a similar conformation with identical enzyme-HFG interactions except for the stacking interaction between the quinazoline-4-one moiety of HFG and Phe415 (Fig 3A and 3B).

Structural analysis of *Tg*PRS-L-pro-L95 shows that the binding of L-pro induces the movement of the side chain of Arg470 which forms a salt bridge between the carboxylic group of L-pro and the guanidium group of Arg470 (S6 Fig). This consequentially provides ample space to accommodate the binding of L95 in the ATP pocket–providing a good rationale as to why L95 shows tight binding in the presence of L-pro as indicated by T_m_ measurements. Superimposition of *Tg*PRS-HFG-L95 and *Tg*PRS-HFG-ANP shows that the ATP binding loop (residues 470–483) is largely displaced and significant side-chain deviations were found for Glu472 and Arg481 (Fig 3B). This displacement is indicative of an accommodative effort for the phenylacetamide moiety of L95 by the active site of the enzyme. Differential loop movements induced by ligand binding (528–538, particularly Phe534-Ala535-Gly536 where in Phe534 adopts unique rotameric conformations) were observed within HFG-ANP bound, HFG-L95 bound and apo *Tg*PRS enzyme when compared to the L-pro-L95 bound structures (S7 Fig). Similar loop movements and side-chain conformation flips were also found between *Hs*PRS-HFG-ANP (PDB ID: 4HVC) and *Hs*PRS-ATP pocket ligand (PDB Id: 7OT3)[21,36]. The HFG binding loop (residues 405–420) is found to be disordered in almost all HFG bound structures (Fig 3B).

The above ligand-induced structural movements of loops are coherent with the differential ΔT_m_ values reported above. The similarity in ΔT_m_ values of *Tg*PRS-HFG-L95 and *Tg*PRS-L-pro-L95 suggest that the binding mode of L95 in the presence of L-pro mirrors that of L95 with HFG. However, the higher ΔT_m_ value for *Tg*PRS-HFG-ANP reflects a more stable association of HFG with ATP than HFG with L95 or L95 with L-pro. Within the ternary complexes, the inhibitors/substrates contact each other peripherally and the interface area for HFG-L95, L-pro-L95 and, HFG-ANP are 27, 35, and 77 Å^2^ respectively (S8 Fig and S1 Table). The significantly higher surface interface overlap between HFG and ANP in the complex is likely to be due to the phosphate groups between HFG and the adenine ring of ANP.

### Cell-based drug gradient assays indicate synergism between HFG and L95

**Fig 4:**
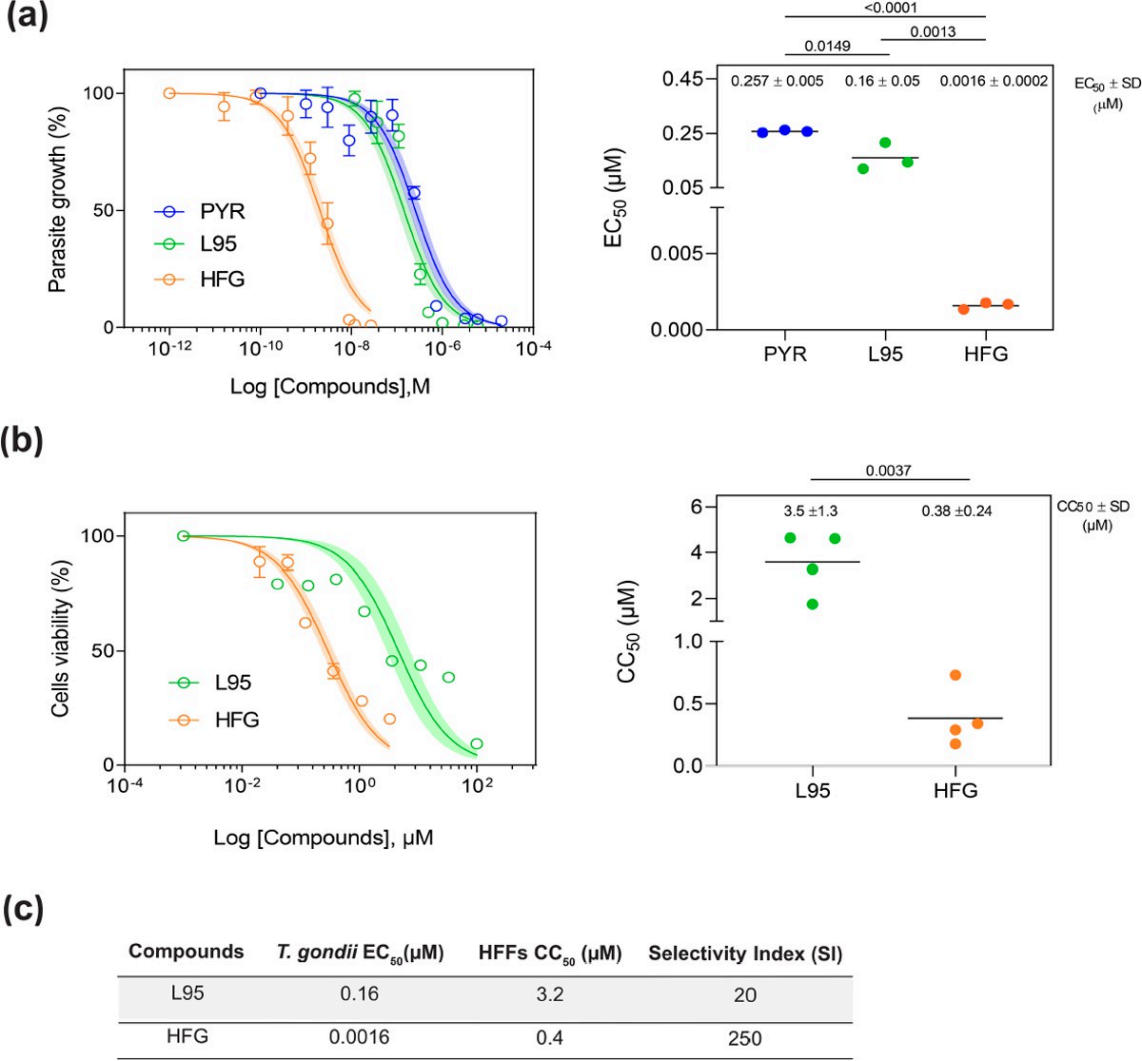
Cell-based inhibition profiles of HFG and L95 against parasite and host cells. (a) Graphs showing the dose-response curves (left panel) and the EC_50_ values (right panel) for inhibition of *T*. *gondii* growth *ex vivo* in response to increasing concentration of pyrimethamine, L95 or HFG. Confluent HFF monolayer was infected with tachyzoites of *T*. *gondii* RH strain expressing the NanoLuc luciferase (RH NLuc). EC_50_ values of each biological replicate were determined by non-linear regression analysis. EC_50_ data are presented as mean ± SD from at least 2 independent biological replicates, each with 3 technical replicates. (b) Effect of L95 and HFG on host-cell viability. Dose-response curves (left panel) and CC_50_ values (right panel) of HFFs in presence of L95 or HFG compounds are shown. HFF cells were incubated for 72 h in the presence of increasing concentrations of the indicated compounds. Cell viability was revealed using the CellTiter-Blue assay kit (Promega) and the CC_50_ was determined by non-linear regression analysis. Dose-response graphs represent the mean ± SD of 3 technical replicates from one experiment. Shaded error envelopes depict 95% confidence intervals. EC_50_ and CC_50_ values are the mean ± SD from 4 independent biological replicates, each with 3 technical replicates. Statistical analyses were performed using one-way ANOVA test. (c) SI values were calculated by dividing the human cells CC_50_ with the *T*. *gondii* EC_50_.

**Fig 5:**
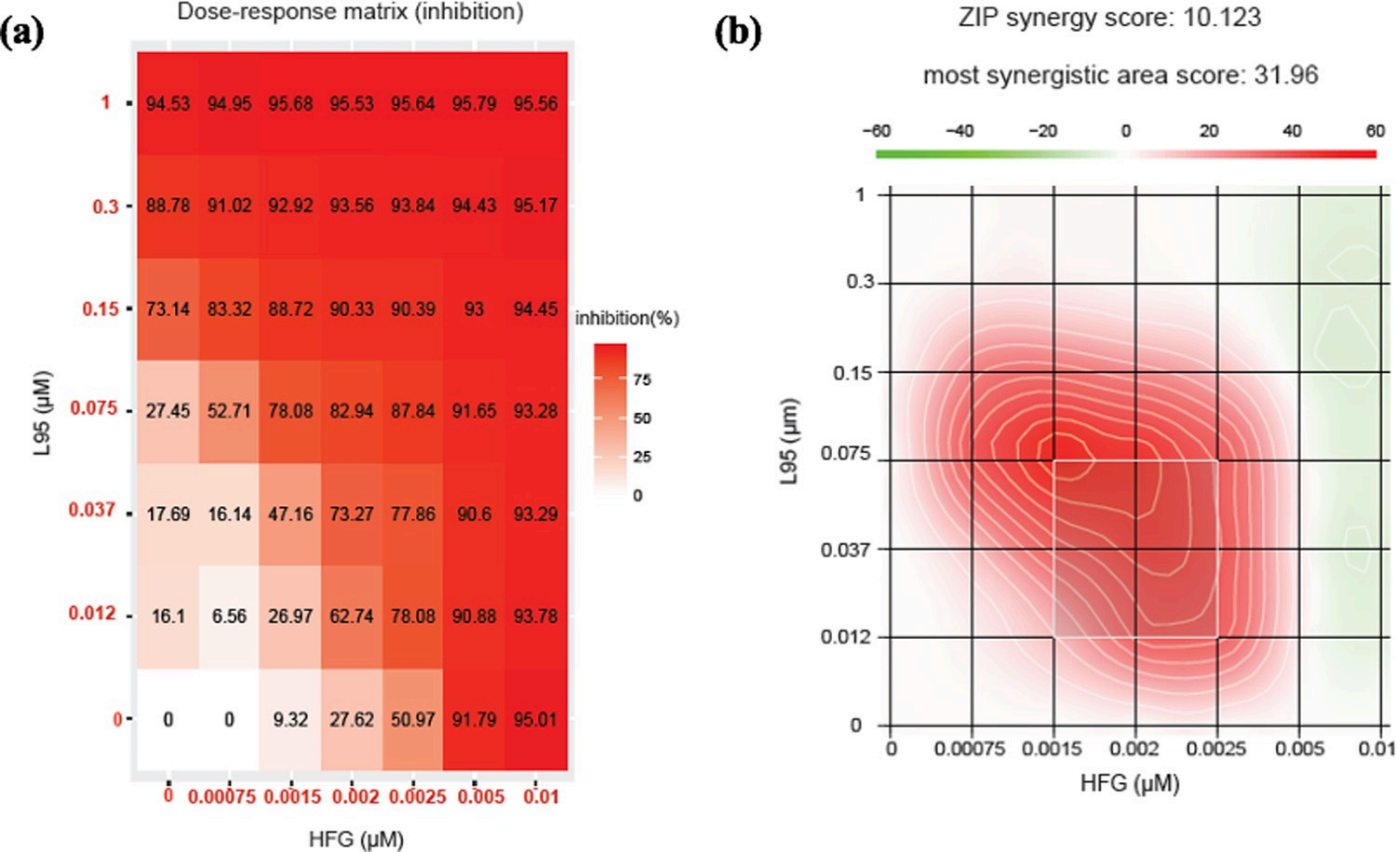
Combinational inhibition profile of HFG and L95 against parasite cell-based system. **(a)** Dose-response matrix of L95 and HFG on RH NLuc *T*. *gondii* parasites after 48 h of growth in presence of the indicated concentrations of each compound. The concentration gradient for HFG used was 0 to 0.01 μM and for L59 was 0 to 1 μM. Percentages of growth inhibition were determined based on measured luminescence. **(b)** Synergy landscape showing synergistic and antagonistic dose regions in red and green respectively, as determined using the Synergy Finder application with the ZIP reference model. The synergy score for a drug combination is averaged (10.123) over all the dose combination measurements (ZIP synergy score). The most synergistic area (score of 31.96) is delimited by a white square.

To explore the anti-parasitic effects of L95 and HFG drug combination, an *ex vivo* cell-based assay was done to monitor the intracellular growth of *Toxoplasma gondii* (*T*. *gondii*) tachyzoites through expression of NanoLuc luciferase (RH NLuc) in a human foreskin fibroblast (HFF) host-cell monolayer [47]. Firstly, the efficiency of L95 and HFG were determined in single drug assays using pyrimethamine as reference for parasite growth inhibition (Fig 4A). In these assays, both L95 and HFG inhibited parasite proliferation at nM concentrations with measured half maximum effective concentration (EC_50_) of 160 and 1.6 nM respectively, which is more efficient than the pyrimethamine positive control (EC_50_ 257 nM) (Fig 4A). In parallel, we evaluated the cytotoxicity of these compounds against HFFs and determined a selectivity index (SI). The HFFs half-maximal cytotoxicity concentration (CC_50_) for L95 and HFG was calculated to be 3.2 μM and 0.4 μM with a selectivity index (SI) of 20 and 250 respectively. The SI was calculated by comparing the *T*. *gondii* EC_50_ to the CC_50_ of HFF cells used for *Toxoplasma* cultures (Fig 4B and 4C). These data indicate that L95 has a parasite selectivity, which is intriguing considering its inhibitory effectiveness against *Hs*PRS enzyme (See Figs 1C, 1D and 4). To investigate the combinatorial effects of L95 and HFG against *T*. *gondii*, we performed a multi-drug combination profiling analysis of parasite growth inhibition. The observed drug combination responses (the input dose-response matrix can be seen in Fig 5A) were compared with expected responses calculated by means of the Zero Interaction Potency (ZIP) synergy reference model using the SynergyFinder web-application to identify any synergistic/additive effects [48,49]. The ZIP score measures synergy by comparing the change in potency of dose-response curves between individual drugs and their combination [48,49]. Fig 5B shows an overall additive score of 10.12 with a synergistic area score of 31.96 for L95 concentrations ranging from 12 to 75 nM in combination with HFG ranges of 1.5 to 2.5 nM. Together these results indicate the potency of L95 and HFG inhibitor combination to inhibit *T*. *gondii* growth and reveal an overall additive effect when used in combination.

## Discussion

Aminoacyl-tRNA synthetases (aaRSs) have over the last decade evolved as effective, novel and specific targets for many disease conditions [16,20,21,24,31,32,45]. The aaRS inhibitors are now also being used as scaffolds for anti-oncogenic drug development [34,35]. Inhibition of these enzymes stalls protein translation thus providing a roadblock in the further development of the parasite or diseased cell [16,20,31,32,45,50,51].

Here, we tested two inhibitors with distinct mechanisms of action. L95 and HFG act wherein the former inhibits PRS activity by binding to the ATP site while the latter binds to the L-pro and tRNA binding sites. Therefore, the prospective therapeutic combination of HFG and L95 occupies all three substrate sites, rendering the target completely blocked and inactive. The study highlights an ‘ortho-steric cocktail’–a strategy of simultaneous targeting of multiple pockets within the active site by two different ligand scaffolds. The thermal shift profile of L95 in presence of L-pro implies a strong complex. Further, both *Tg*PRS-L-pro-L95 and *Tg*PRS-HFG-L95 complexes have similar thermal profiles (Fig 1B). Interestingly, the *Tg*PRS-HFG-ATP complex has a higher ΔT_m_ value (37.6°C) than those of *Tg*PRS-L-pro-L95 (20.7°C) and *Tg*PRS-HFG-L95 (23.6°C) (Fig 1B). This is most likely because of the ligand induced movement of loops and consequent differences in the side chain conformation (particularly Arg470 and Phe534).

The potencies of L95 and HFG against *Toxoplasma* parasites and *Tg*PRS enzyme were validated via enzymatic and cell-based assays. Both IC_50_ and EC_50_ data revealed that these drugs are effective at nM concentrations when used individually (Figs 1C, 1D and 4). In context of selectivity, HFG was more selective against *T*.*gondii* parasite and the parasitic enzyme than L95 (cellular SI of HFG = 250 vs cellular SI of L95 = 20; enzymatic SI of HFG = ~2 vs enzymatic SI of L95 = ~1). In thermal stability, the *Tg*PRS-HFG-L95 combination had a higher ΔT_m_ value than each ligand alone (23.6°C compared to 8.1°C for *Tg*PRS-HFG and 17.8°C for *Tg*PRS-L95), suggesting that when used in tandem, they could improve *Tg*PRS inhibition in an additive fashion (Figs 1B and S2). When the cell-based drug-combination assay was performed using a 7x7 matrix, we discovered that these compounds act synergistically when used in a specific range at low doses (see the most synergistic area with a high synergy score of 31.96; Fig 5). However, the overall synergy score of ~10 proves that the inhibitor combination is more likely to have a general additive effect. Indeed, HFG is more active than L95, and we cannot rule out the possibility that if HFG is used at higher concentrations, its effect would mask L95 activity. The similarities observed in the structures of *Tg*PRS-L-pro-L95 and *Tg*PRS-HFG-L95 indicate that the potency of L95 remains unaffected in presence of either L-pro or HFG, which would be consistent with an additive activity at concentrations close to the IC_50_. This is in contrast to the cooperative binding of ligands thus indicating a gap in our understanding of the structural machinations of this approach.

The authors understand the limitations of dual targeting using these exact scaffolds and the caveats that come along with it for bradyzoite targeting, which remains critical for *Toxoplasma* targeting. This exact therapeutic combination might not be of ultimate utility considering the similarity in PK properties and potential toxicity of both these compounds. Bioavailability and cost feasibility would remain a critical bottleneck for the actual medical translation of this combination therapy. Furthermore, as far as the possibility of off-site targets by ATP-mimetics is concerned–previously characterized inhibitors have given ample evidence for the highly specific nature of ATP recognition by enzymes and why such an occurrence is rare [16]. Here we provide proof that two ligand moieties can simultaneously occupy all catalytic pockets of an essential aaRS enzyme (here, *Tg*PRS). Indeed, our approach can be extended to other enzyme-drug systems where novel assays screen for potency as well as synergy. Drug targeting of proteins has so far relied on finding single inhibitors of high potency but our novel data show that it is feasible to design two (or more) inhibitors that may simultaneously attack any available druggable pockets in target proteins. Borrelidin is a natural product and is an example of a simultaneous multi-site aaRS inhibitor. Designing and producing such molecules synthetically has many caveats including but not limited to selectivity and cost feasibility. The future prospects of such and other multi-site inhibitors remains to be explored in a systematic manner. Our approach detailed here will allow rescreening of even known enzyme-drug combinations with new compound libraries in order to discover synergistic inhibitors that can co-occupy and co-inhibit the same target enzyme.

## Materials and methods

### Protein purification

*Tg*PRS was produced in an *E*.*coli* BL-21 strain in accordance with methods published previously [20,45]. Briefly, protein expression was induced by adding 0.5 mM isopropyl β-D-thiogalactopyranoside (IPTG) to cells grown at 37°C for 4 h, and for 20 h post-induction at 18°C. The cells were harvested by centrifugation at 5,000 g for 15 min. The bacterial pellet was suspended in a buffer containing 50 mM Tris–HCl pH 8.0, 200 mM NaCl, 3 mM ßME, 15% v/v glycerol, 0.1 mg ml^-1^ lysozyme and EDTA free protease inhibitor cocktail (Roche). The cells were lysed by sonication and cleared by centrifugation at 20,000 g for 45 min. The cleared supernatant with maltose-binding protein (MBP) and 6xHis tagged proteins were applied to NiNTA beads (GE Healthcare) and protein was eluted with buffer 50 mM Tris–HCl pH 8.0, 200 mM NaCl, 10 mM ßME and 250 mM Imidazole. The eluted protein fractions were dialyzed against 30 mM HEPES pH 7.5, 20 mM NaCl, 1 mM DTT and 0.5 mM EDTA (buffer A). The protein was further purified by heparin chromatography (GE Healthcare) using NaCl gradients with buffer B containing 30 mM HEPES pH 7.5, 500 mM NaCl, 1 mM DTT and 0.5 mM EDTA. The protein peak was found at 40% buffer B. The MBP and 6xHis tag was removed by incubating with TEV protease at 20°C for 24 h. The cleaved *Tg*PRS protein was concentrated using 10 kDa cut-off Centricon centrifugal device (Millipore) and purified by gel filtration chromatography on a Superdex 200 column 16/60 GL (GE Healthcare) equilibrated with 20 mM HEPES pH 7.5, 200 mM NaCl and 2 mM DTT. Bovine serum albumin (66 kDa, Sigma) was used as a standard for the molecular mass estimation. The eluted fractions were checked by SDS-PAGE and the pure fractions were pooled, concentrated in 50 mM Tris–HCl pH 8.0, 200 mM NaCl, 10 mM ßMe and stored at -80°C.

### Thermal shift assays

Thermal shift assays were performed for *Tg*PRS and *Hs*PRS in the presence of substrates (L-pro and ATP) and inhibitors (L95 and HFG) as per previous protocol [20,45]. Briefly, the purified enzymes (1 μM) alone and/or with substrates/inhibitors (2 mM/50 uM) were heated from 25 to 99°C at a rate of 1.2°C min^-1^ and fluorescence signals of the SYPRO orange dye was monitored by a quantitative real-time PCR system (Life Technologies). Note that titration experiments determined that saturation of active pocket enzymes was obtained at 1:50 protein: compound ratio (S2B Fig). The melting curve is an average of three measurements and data were analysed using Protein Thermal shift software (v1.3, Thermofisher). The inhibitors and substrate alone in assay buffers, along with no enzyme controls were used and flat lines were observed for these fluorescence readings across the temperatures. The derivative T_m_ was used for analysis.

### Enzyme inhibition assays

These were done using malachite green assay and the assay was performed according to the earlier published report [20]. Briefly, the reaction was observed for 25 μM ATP, 25 μM L-pro and 400 nM recombinant PRS enzyme in a buffer containing 30 mM HEPES (pH 7.5), 140 mM NaCl, 30 mM KCl, 50 mM, MgCl_2_, 1mM DTT and 2 U/ml E. coli inorganic pyrophosphatase (NEB) at 37°C. The enzymatic reactions (100 μl total volume) were performed in a clear, flat-bottomed, 96-well plates (Costar 96-well standard microplates). The reaction mixture was incubated for 40 min at 37° C. The reaction was stopped by adding 25 μl of malachite green dye solution to the reaction mixture and levels of inorganic phosphate (Pi) were detected after incubation of 5 min at room temperature. Absorbance was measured at 630 nm using a Spectramax M2 (Molecular Devices). Reactions without PRS enzyme were performed as background controls, values of which were subtracted from the reactions with enzyme. HFG and L95 were added to the aminoacylation assay reaction buffer in varying concentrations ranging from 50 to 0.005 μM. The IC_50_ values for the data is shown for three replicates as the mean ± SD.

### Crystallization and structure determination

Purified *Tg*PRS enzyme was used for crystallization via the hanging-drop vapour-diffusion method at 20°C using commercially available crystallization screens (Index, JCSG^+^, Morpheus, PACT premier, PGA, Crystal Screen, PEG/Ion and ProPlex (Hampton Research and Molecular Dimensions). Initial screening was performed in 96-well plates using a nano drop dispensing Mosquito robot (TTP Labtech). Three different drop ratios were used for the crystallization trials by mixing 75, 100 or 50 nl purified protein solution with 75, 50 or 100 nl reservoir solution, respectively (i.e., 1:1, 2:1 and 1:2 drop ratios). Each of the drops was equilibrated against 100 μl of the corresponding reservoir solution. Before crystallization, 1 mM L95 and 2 mM L-pro were added to *Tg*PRS in one set up and with HFG in another while the mixtures were incubated at 4°C for 10 min. Diffraction quality crystals of ternary complexes were obtained at 20°C by the hanging-drop vapour-diffusion method using 50 nl each of the protein mixture solutions and 50 nl crystallization well solution. The *Tg*PRS-L-pro-L95 crystals were obtained in B5 of JCSG^+^ screen (0.1 M sodium cacodylate pH 6.5 and 40% v/v MPD 5% w/v PEG 8000). The *Tg*PRS-HFG-L95 crystals were obtained in Morpheus G2 screen [0.1 M carboxylic acids (0.2 M sodium formate, 0.2 M ammonium acetate, 0.2 M sodium citrate tribasic dihydrate, 0.2 M potassium sodium tartrate tetrahydrate, 0.2 M sodium oxamate), 0.1 M buffer pH 6.5 (imidazole; MES monohydrate (acid) and 30% v/v precipitant mix (40% v/v Ethylene glycol; 20% w/v PEG 8000)]. Plate/rod-shaped crystals appeared within 10 days. The crystals were mounted in nylon loops (Hampton Research) or litho loops (Molecular Dimensions) after being soaked for 10–30 s in a cryoprotectant containing the corresponding crystallization mother liquor with 20% (v/v) glycerol. The crystals were subsequently flash-cooled in liquid nitrogen. The X-ray diffraction data sets were collected on beamline I03 at Diamond Light Source (DLS), United Kingdom at a wavelength of 0.976 Å for the *Tg*PRS-HFG-L95 structure while for the *Tg*PRS-L-pro-L95 structure at PROXIMA-1, SOLEIL, France at a wavelength of 0.979 Å. The data were processed by the Xia2 auto-processing pipeline using DIALS for integration [52]. The initial models were determined by the molecular-replacement (MR) method using Phaser [53] with A chain of *Tg*PRS-HFG-ANP (PDB ID 5XIQ) without bound ligands (HFG and ANP) as the template. The structure was further refined by iterative cycles of refinement with Phenix [54] and model building with COOT [55]. The final models were refined to 1.7 Å resolution with R_work_/R_free_ values of 15.3/18.1 and 19.8/23.0%, respectively for ternary complexes *Tg*PRS-L-pro-L95 and *Tg*PRS-HFG-L95. The stereo-chemical quality of the models was assessed and corrected using MolProbity [56]–finally showing good geometry quality with 98% residues in most favoured regions of the Ramachandran plot for the *Tg*PRS-L-pro-L95 and *Tg*PRS-HFG-L95 complexes, respectively. Statistics of data collection and structure refinement are given in Table 1. The atomic coordinates and structure factors of *Tg*PRS-L-pro-L95 and *Tg*PRS-HFG-L95 complexes have been deposited in the Protein Data Bank (PDB) with the accession codes 7EVV and 7EVU, respectively. Surface areas for the protein and ligand molecules were computed using the PDBePISA server [57]. The figures were prepared using Chimera [58] and PyMOL [59].

### Measurement of EC_50_ for *Toxoplasma gondii* parasites

A confluent Human Foreskin Fibroblast (HFF) monolayer was infected with 2,000 tachyzoites of RH parasites expressing the NLuc luciferase (RH NLuc) for 2 h. After parasites invasion, each drug was added to the culture medium. After 48 h of incubation at 37°C, the medium was replaced by 50 μL of PBS. The luminescence assay was performed using a Luciferase Assay System according to manufacturer’s instructions (Promega). After 3 min of incubation, luminescence was measured using a (BMG Labtech) plate reader. EC_50_ were determined using non-linear regression analysis of normalized data and assuming a sigmoidal dose response. EC_50_ values for each compound represent an average of three independent biological replicates. Statistical analyses were performed using one-way ANOVA test with the GraphPad software.

### Measurement of CC_50_ for human cells and determination of selectivity index

10,000 HFFs were plated in 96-well plates for 1 h and incubated with exponential concentrations of indicated compounds in a final volume of 100 μL. After 72 h of culture the CellTiter-Blue dye (Promega) (20μL/well) was added directly to each well. The plates were then incubated at 37°C for 2 h to allow cells to convert resazurin to resorufin before recording fluorescence (560(20) Ex/590(10) Em) with a (BMG Labtech) plate reader. Human cells cytotoxicity concentration (CC_50_) was determined using non-linear regression curve of normalized data. The CC_50_ values represent the average of four biological experiments. Selectivity Index (SI) was obtained by the average of the human CC_50_ divided by the average of *T*. *gondii* EC_50_.

### Gradient combinational assay

Drug dilutions used to determine the dose-response curves were utilized to predict the drug combination response. We defined a 7x7 dose matrix (% inhibition range between 0 and 100) on 96-well plates. Confluent HFFs monolayer was infected with RH NLuc parasites for 2 h before adding drugs combination. After 48 h of culture the medium was replaced by PBS and the luminescence was read as described above. The overall synergy score for a drug pair is calculated based on Zero Interaction Potency (ZIP) reference model using SynergyFinder web tool. Deviations between predicted and expected responses with positive and negative score denote synergism and antagonism, respectively [48,49]. When synergy score is < -10, the interaction between two drugs is likely to be antagonistic; from -10 to 10, the interaction between two drugs is likely to be additive; and >10, the interaction between two drugs is likely to be synergistic. The most synergistic area represents the most synergistic 2-by-2 dose-window in a dose-response matrix.

## Supporting information

S1 Fig (a) Chemical structure of ligand L95 and its functional groups. (b) Structural overlay of L95 with ATP.
(TIFF)

S2 Fig TSA Melt Curves (a) *Tg*PRS melt curves with different combinations of inhibitors and substrates. (b) *Tg*PRS differential ratios for saturation of protein. (c) *Hs*PRS melt curves with different combinations of inhibitors and substrates.
(TIFF)

S3 Fig The difference Fourier map (Fo-Fc) of the bound ligands in the aminoacylation site of chain B of *Tg*PRS-HFG-L95 complex. The Fo-Fc maps are contoured at 3 σ and shown as green mesh representations. Ligands L95 (yellow), HFG (pink) are shown as sticks.
(TIFF)

S4 Fig Important interactions between ligand L95 with protein residues and water molecules. Ligand L95 (yellow) and protein residues (purple) are shown as sticks representations. The water molecules are shown as red spheres. Hydrogen bonds are shown as black dashed lines.
(TIFF)

S5 Fig The 6-methylpyridine ring of ligand L95 sandwiched between Arg594 and Phe485 via π-π stacking interaction (~3.9 Å). (TIFF)

S6 Fig Overlay of L-pro-L95 (purple) bound structures with apo form of *Tg*PRS (pink) showing the different conformations of active site residues Arg470, Arg594 and F534, and the ATP binding loop. (TIFF)

S7 Fig Overlay of apo (pink), L-pro-L95 bound (purple), HFG-L95 bound (steel blue) and HFG-ANP bound (olive) *Tg*PRS structures showing the displacement of the loop residues 528–538. (TIFF)

S8 Fig Molecular surface representation showing the association of bound ligands in ternary complexes of *Tg*PRS-L-pro-L95, *Tg*PRS-HFG-L95 and *Tg*PRS-HFG-ANP. The *Tg*PRS surface is displayed as 70% transparent surface and the bound ligands surface are shown as solid surfaces.
(TIFF)

S1 Table Buried surface areas of the inhibitors and substrates within *Tg*PRS. (XLSX)
