# Supplementary figures and images for: Double drugging of prolyl-tRNA synthetase provides a new paradigm for anti-infective drug development
Source: PLoS Pathog. 2022 Mar 25;18(3):e1010363. doi: 10.1371/journal.ppat.1010363 (PMC9004777; doi:10.1371/journal.ppat.1010363)

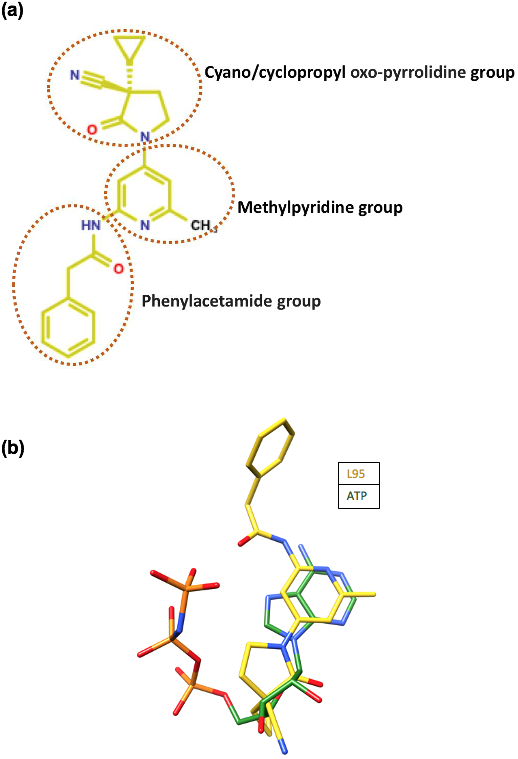

Supplement: S1 Fig — (a) Chemical structure of ligand L95 and its functional groups. (b) Structural overlay of L95 with ATP. (TIFF) [file ppat.1010363.s001.tiff]

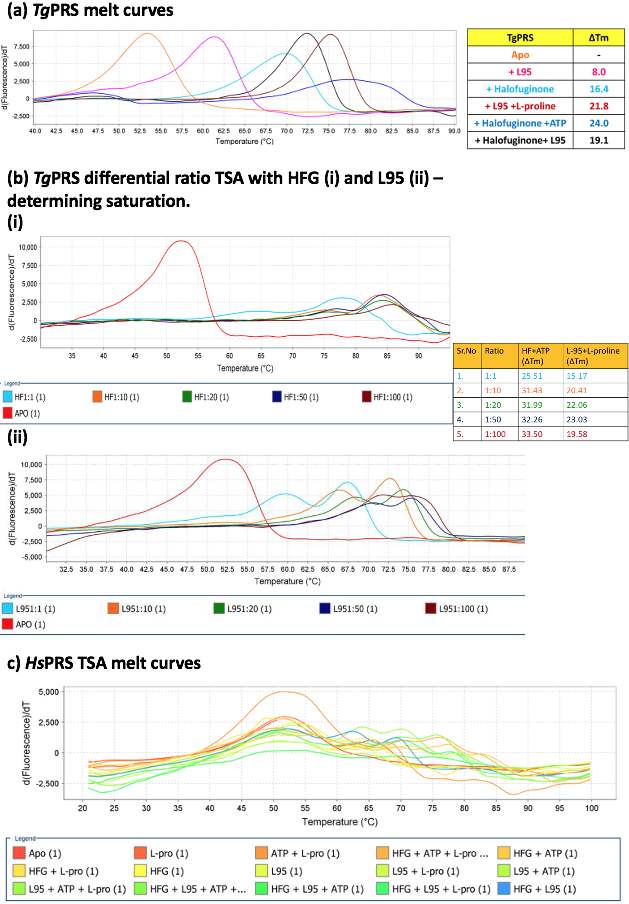

Supplement: S2 Fig — TSA Melt Curves (a) TgPRS melt curves with different combinations of inhibitors and substrates. (b) TgPRS differential ratios for saturation of protein. (c) HsPRS melt curves with different combinations of inhibitors and substrates. (TIFF) [file ppat.1010363.s002.tiff]

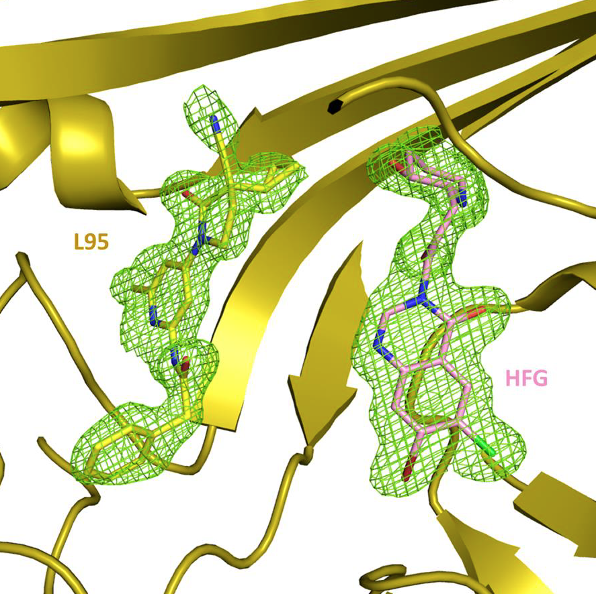

Supplement: S3 Fig — The Fo-Fc maps are contoured at 3 σ and shown as green mesh representations. Ligands L95 (yellow), HFG (pink) are shown as sticks. (TIFF) [file ppat.1010363.s003.tiff]

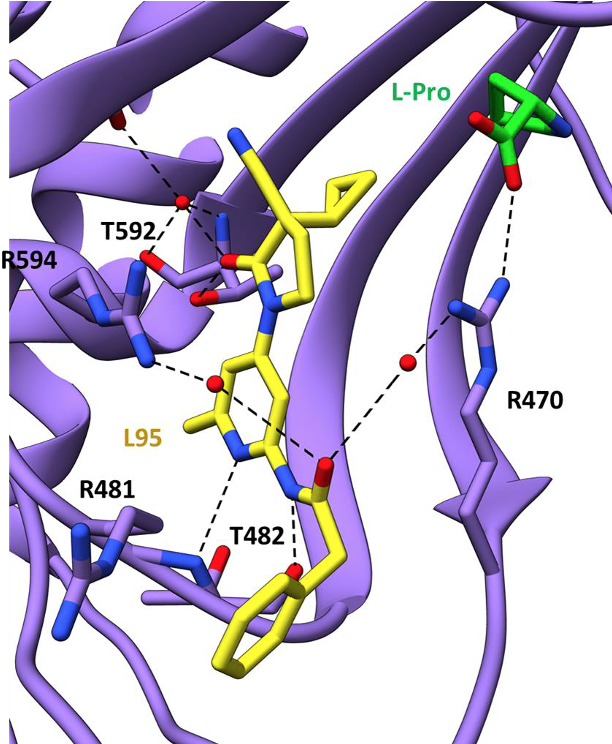

Supplement: S4 Fig — Ligand L95 (yellow) and protein residues (purple) are shown as sticks representations. The water molecules are shown as red spheres. Hydrogen bonds are shown as black dashed lines. (TIFF) [file ppat.1010363.s004.tiff]

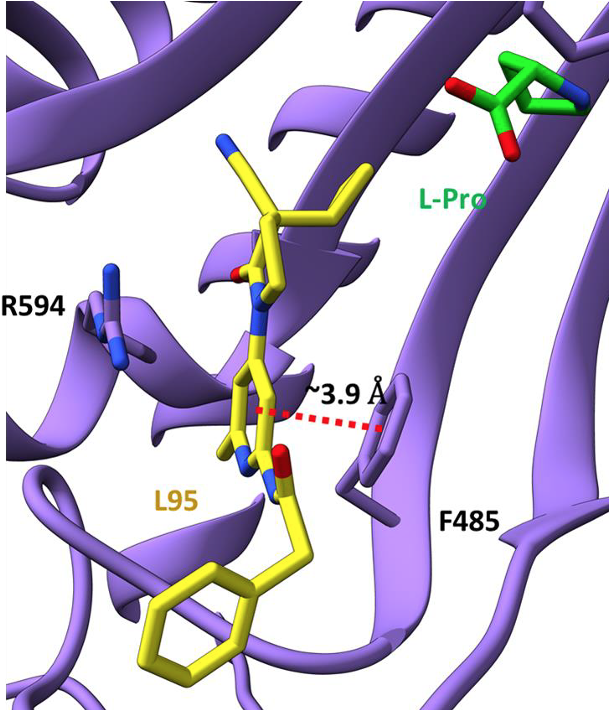

Supplement: S5 Fig — (TIFF) [file ppat.1010363.s005.tiff]

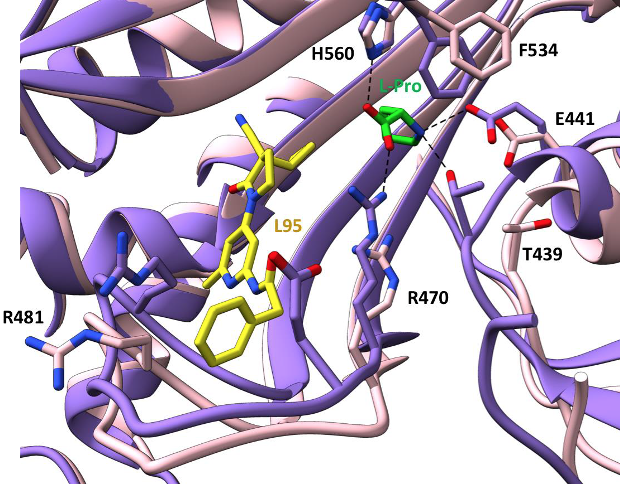

Supplement: S6 Fig — (TIFF) [file ppat.1010363.s006.tiff]

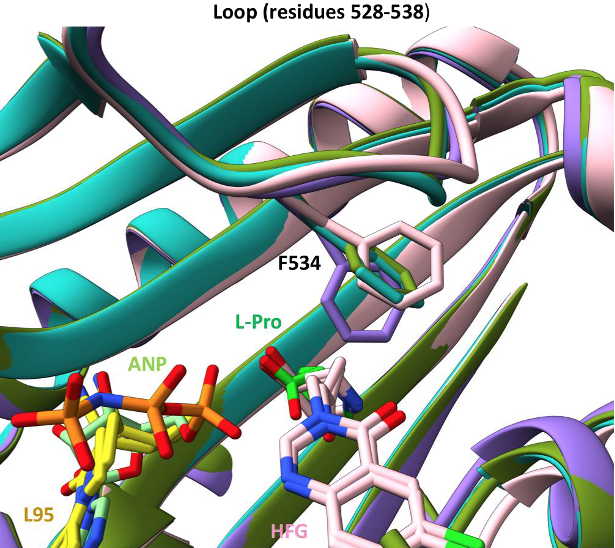

Supplement: S7 Fig — (TIFF) [file ppat.1010363.s007.tiff]

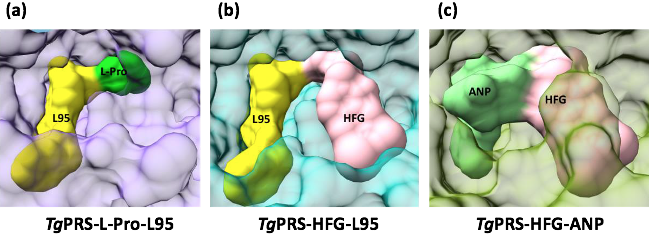

Supplement: S8 Fig — The TgPRS surface is displayed as 70% transparent surface and the bound ligands surface are shown as solid surfaces. (TIFF) [file ppat.1010363.s008.tiff]
